# Utilizing transcriptomics and metabolomics to unravel key genes and metabolites of maize seedlings in response to drought stress

**DOI:** 10.1186/s12870-023-04712-y

**Published:** 2024-01-08

**Authors:** Yipu Li, Zhijun Su, Yanan Lin, Zhenghan Xu, Haizhu Bao, Fugui Wang, Jian Liu, Shuping Hu, Zhigang Wang, Xiaofang Yu, Julin Gao

**Affiliations:** 1 Region Research Center for Conservation and Utilization of Crop Germplasm Resources in Cold and Arid Areas, Agricultural College, Inner Mongolia Agricultural University, Hohhot, China; 2 Vocational and Technical College, Inner Mongolia Agricultural University, Baotou, China

**Keywords:** Maize seedlings, Drought stress, Transcriptome analysis, Metabolomics, Differential genes and metabolites

## Abstract

**Background:**

Drought stress can substantially restrict maize growth and productivity, and global warming and an increasing frequency of extreme weather events are likely to result in more yield losses in the future. Therefore, unraveling the molecular mechanism underlying the response to drought stress is essential for breeding drought-resilient crops.

**Results:**

In this study, we subjected the 3-leaf-period plants of two maize inbred lines, a drought-tolerant line (si287) and a drought-sensitive line (X178), to drought stress for seven days while growing in a chamber. Subsequently, we measured physiological traits and analyzed transcriptomic and metabolic profiles of two inbred lines. Our KEGG analysis of genes and metabolites revealed significant differences in pathways related to glycolysis/gluconeogenesis, flavonoid biosynthesis, starch and sucrose metabolism, and biosynthesis of amino acids. Additionally, our joint analysis identified proline, tryptophan and phenylalanine are crucial amino acids for maize response to drought stress. Furthermore, we concentrated on tryptophan (Trp), which was found to enhance tolerance via IAA-ABA signaling, as well as SA and nicotinamide adenine dinucleotide (NAD) consequent reactive oxygen species (ROS) scavenging. We identified three hub genes in tryptophan biosynthesis, indole-3-acetaldehyde oxidase (*ZmAO1*, 542,228), catalase 1 (*ZmCAT1*, 542,369), and flavin-containing monooxygenase 6 (*ZmYUC6*, 103,629,142), High expression of these genes plays a significant role in regulating drought tolerance. Two metabolites related to tryptophan biosynthesis, quinolinic acid, and kynurenine improved maize tolerance to drought stress by scavenging reactive oxygen species.

**Conclusions:**

This study illuminates the mechanisms underlying the response of maize seedlings to drought stress. Especially, it identifies novel candidate genes and metabolites, enriching our understanding of the role of tryptophan in drought stress. The identification of distinct resistance mechanisms in maize inbred lines will facilitate the exploration of maize germplasm and the breeding of drought-resilient hybrids.

**Supplementary Information:**

The online version contains supplementary material available at 10.1186/s12870-023-04712-y.

## Background

Due to ongoing global climate change, agricultural production worldwide is expected to be heavily inflicted by droughts [1]. Furthermore, the growing global population will lead to a 30% increase in water demand for agriculture by 2025, and if current levels of water consumption remain unchanged, almost half of the world’s population will suffer severe water stress by 2030 [2]. To address these challenges, it is crucial to develop strategies that can mitigate the impacts of drought. Breeding drought-resilient crops is considered one of the most cost-effective and efficient measures to achieve this goal [3]. Understanding the responses of different crops to drought will lay the cornerstone towards breeding drought-resilient varieties. Recent studies have highlighted that maize plants undergo significant changes in gene expression and alternative splicing when exposed to drought stress. *ZmMPKL1* positively regulates seedling drought tolerance by controlling the expression of abscisic acid (ABA) biosynthetic and catabolic genes [4]. *ZmDREB2.7, ZmVPP1 and ZmNAC111* regulate maize seedling drought tolerance conferring inducible expression [5–7]. These maize drought tolerant genes have been discovered by a few easy-to-measure traits, such as, survival rate. However, our understanding of the complex biochemical and physiological responses that occur in maize plants during drought stress is limited.

The advent of metabolomic technologies has greatly aided our understanding of the physiological and metabolic pathways underlying crop responses to drought stress. Metabolite profiling studies have revealed that drought stress triggers the production of a diverse range of metabolites in maize. In response to drought, maize undergoes various adaptive reactions, including the synthesis of sugars and free amino acids to regulate osmotic pressure, the activation of anti-oxidative enzymes such as superoxide dismutase (SOD) and catalase (CAT) [8], and the production of antioxidants like glutathione s-transferase (GSH-S) [9] and flavonoids [10] to counteract reactive oxygen species (ROS) or regulate stomatal opening and closing via ABA hormone signaling [11]. The metabolic pathways of redox homeostasis, osmoregulation, and membrane remodeling play crucial roles in maize drought resistance mechanisms [12]. Additionally, drought stress leads to the accumulation of two photorespiratory amino acids, glycine and serine, in maize [13, 14]. Secondary metabolites derived from tryptophan play vital roles in promoting plant survival upon abiotic stresses, including drought, salinity, and extreme temperatures [15–17].

In this study, to gain insights into the genetic mechanisms underlying maize’s response to drought stress, we conducted a comprehensive study involving physiological, comparative transcriptomic and metabolomic analyses of seedling maize plants subjected to well-watered (W) and drought-stressed (D) conditions. We identified several key genes and metabolites involved in the drought response of maize. The results will provide novel insights into the transcriptome and metabolome mediated drought adaptation in maize seedlings.

## Materials and methods

### Plant growth conditions

Seeds of maize inbred lines si287 (drought-resistant) and X178 (drought sensitive) were sterilized in 10% (v/v) H_2_O_2_ solution for 30 min, followed by washing with distilled water. Then the sterilized seeds were imbibed in distilled water for 24 h. Five seeds of each line were sown in individual 2.5 L pots filled with sandy soil with a pH of 7.2, bulk density of 1,578 kg.m^− 3^, organic carbon content of 0.23% m/m, and organic matter content of 14.17 g/kg. The pots were kept in a growth chamber at Inner Mongolia Agricultural University under controlled environmental conditions of 16/8 light/dark cycle and 60% relative humidity.

### Stress treatments

At the 3-leaf stage, the plants in the pots were subjected to two different watering treatments for a period of 7 days. The first treatment was the well-watered (W) control, in which the plants were watered normally to maintain the soil water content (SWC) at 75%. The second treatment was the drought-stress (D) treatment, in which the plants were irrigated to maintain the soil water content at 45% SWC. The soil water status was monitored every evening to evaluate the soil moisture content. Each treatment was replicated in 8 pots.

### Physiological trait measurements

At the end of the stress period, the weight of dry shoots and dry roots was measured for each plant. Subsequently, we calculated the relative biomass loss and leaf relative water content (RWC) for each plant subjected to both W and D treatments. Leaf RWC was calculated as described in previous studies [18, 19].

### Sample harvesting for total RNA-seq and metabolite profiling

In the D and W treatments, the youngest-leaf tissues were collected from five independent plants to serve as one biological replicate. There were three biological replicates for RNA-Seq analysis and six biological replicates for metabolite profiling. The collected tissue samples were wrapped immediately in aluminum foil and stored at -80℃ until further analysis. Tissue sampling was conducted at the end of the seven-day treatment period.

### Read quality assessment to maize genome and identification of DEGs

The reference genome and gene model annotation files were obtained directly from the genome website. Hisat2 v2.0.5 was used to build an index of the reference genome, and the paired-end clean reads were aligned to the reference genome Zm-B73-REFERENCE-NAM-5.0 (https://ftp.ncbi.nlm.nih.gov/genomes/all/GCF/902/167/145/GCF_902167145.1_Zm-B73-REFERENCE-NAM-5.0/) using the same tool. Hisat2 was chosen as the mapping tool because it can generate a database of splice junctions based on the gene model annotation file, resulting in more accurate mapping compared to other non-splice mapping tools. Fragments per kilobase of exons per million mapped fragments (FPKM) were calculated for each gene based on its length and the number of reads mapped to it. The screening criteria for differentially expressed genes (DEGs) were adjusted *P*_adj_<0.05 and |log2 FoldChange (FC)| >1.

### GO and KEGG enrichment analysis of differentially expressed genes

Gene Ontology (GO) enrichment analysis of DEGs was performed using the clusterProfiler R package with gene length bias corrected. GO terms with a corrected *P* value less than 0.05 were considered significantly enriched by DEGs. KEGG (Kyoto Encyclopedia of Genes and Genomes), a database resource for understanding high-level functions and utilities of biological systems, was used to test the statistical enrichment of DEGs in KEGG pathways. This was also done using the clusterProfiler R package. KEGG provides molecular-level information and is particularly useful for analyzing massive molecular datasets generated by genome sequencing and other high-throughput experimental technologies (http://www.genome.jp/kegg/) [20].

### Metabolite profiling and data analyses

The leaf samples weighing 100 mg each were treated as described in previous studies [21, 22]. ExionLC™ AD system (SCIEX, Framingham, MA, USA) and QTRAP® 6500 + mass spectrometer (Novogene Co. Ltd. Beijing, China) was used to conduct liquid chromatography-mass spectrometry (LC-MS) analyses. Metabolites with > 20% missing values under either W or D conditions were removed followed by a three-step process to eliminate redundant peaks. Each mass feature’s signal intensity underwent a log transformation. Using the R package “pcaMethods”, principal component analysis (PCA) was performed to assess the influence of drought on the maize metabolome [23]. Metabolites were considered differentially accumulated under drought stress conditions when they met the following criteria: (I) Variable Importance for the Projection (VIP) calculated from OPLS-DA ≥ 1 [24]; (II) *P* values from a two-tailed Student’s t test on the normalized peak areas from W and D conditions ≤ 0.05; (III) |Fold Change (D/W) | ≥ 2 [25].

### Combined analysis of genes and metabolites

The pathways enriched with both DEGs and DEMs under the same treatment were identified and visualized on the KEGG pathway map. A histogram was generated to illustrate the level of enrichment for the identified pathways.

### Real-time PCR validation of ABA responsive genes under drought stress

For Real-time quantitative reverse transcription PCR (Real-time PCR) analyses, the first-strand cDNA was synthesized using *TransScript*® First-Strand cDNA Synthesis SuperMix (TransGen Biotech, Beijing, China). Real-time PCR was performed using SYBR Green Mix (TIANGEN, Beijing, China) in a LightCycler96 instrument (Roche, Switzerland), with the maize *GAPDH* gene as the internal reference. The ABA signaling pathway genes *ZmSnRK2.4*, *ZmPP2C-A4*, *ZmPP2C-A7*, *ZmRD17*, *ZmRAB18*, and *ZmLEA* were selected for expression validation using specific primers [25].

### Data analysis

The data are presented as the mean ± standard deviation (SD). Statistical significance (P < 0.05) was determined using one-way analysis of variance (ANOVA) followed by Duncan’s test in SPSS Statistics 26.0 (SPSS Inc., Chicago, IL, USA). All figures were created using Microsoft Office and Adobe Photoshop CC 2017 software.

## Results

### Physiological responses of the two inbred lines to drought stress

**Fig. 1:**
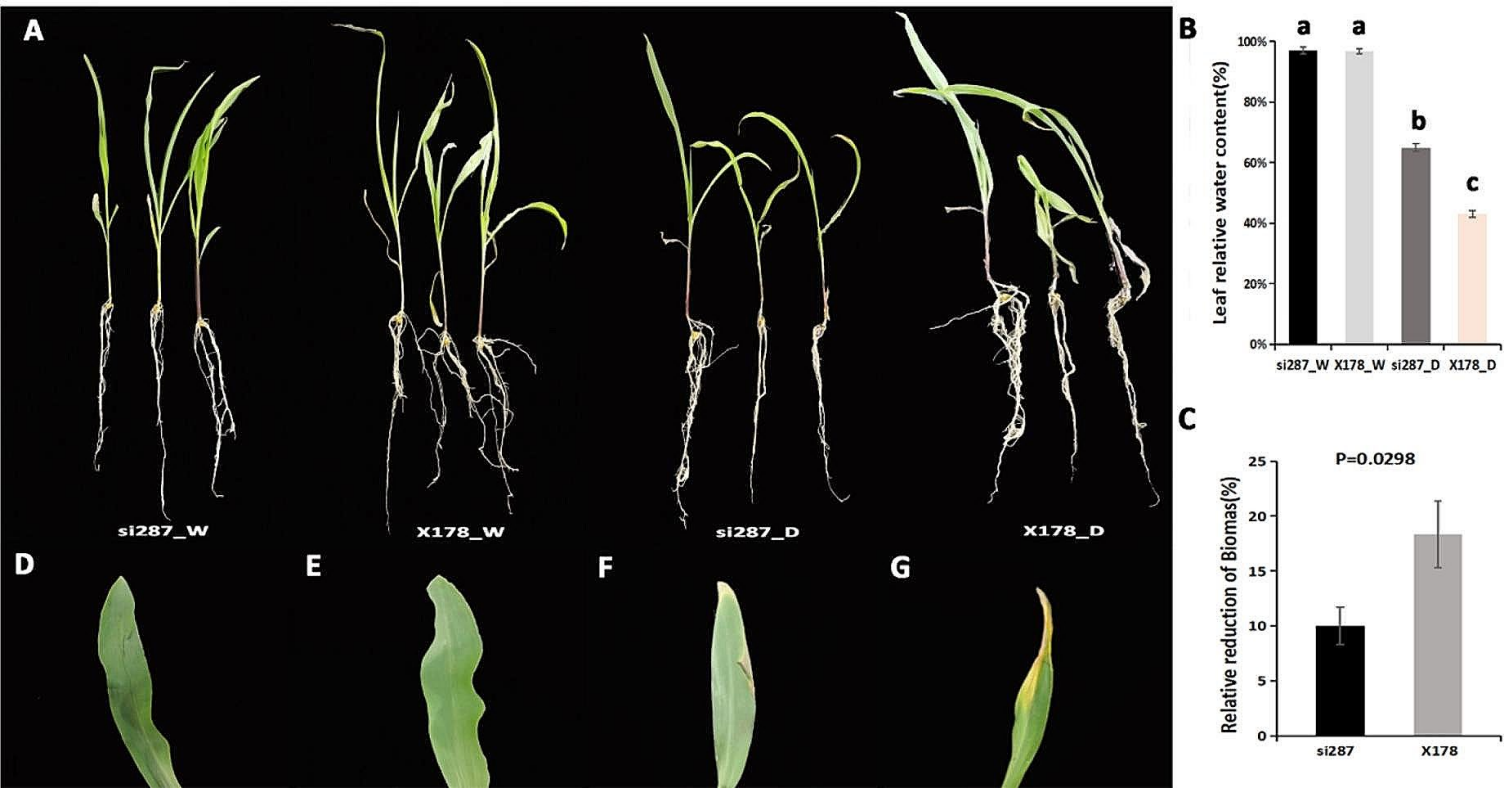
Impact of drought stress on 3-leaf maize. (**A**), Phenotypes of si287 and X178 under well-water (W) and drought stress (D) treatments. (**B**), Leaf RWC (%). (**C**), Relative biomass of 3-leaf-stage plants under drought stress (expressed as a percentage of well-water plant biomass). (**D**) Leaf image of si287_W. (**E**) Leaf image of X178_W. (**F**) Leaf image of si287_D. (**G**) Leaf image of X178_D

Regarding plant morphology (Fig. 1A), plants exposed to drought stress (D) condition showed shorter height with smaller leaf area, lower leaf relative water content (Fig. 1B) and yellow curled leaves compared with the well-water (W) condition (Fig. 1D-G), which reflected nutrient deficiency and flavonoid accumulation [12]. Furthermore, the plant biomass of drought-stressed plants significantly decreased compared to that of well-water treatments (Fig. 1C; Table S1), potentially due to drought-altered gene expression. X178 lost 18.7% of its relative biomass which was 9% higher than that of si287. Overall, these findings suggest that si287 exhibited much stronger resistance to drought stress than X178.

### Comparative transcriptomic analysis of si287 and X178 responses to drought stress

**Fig. 2:**
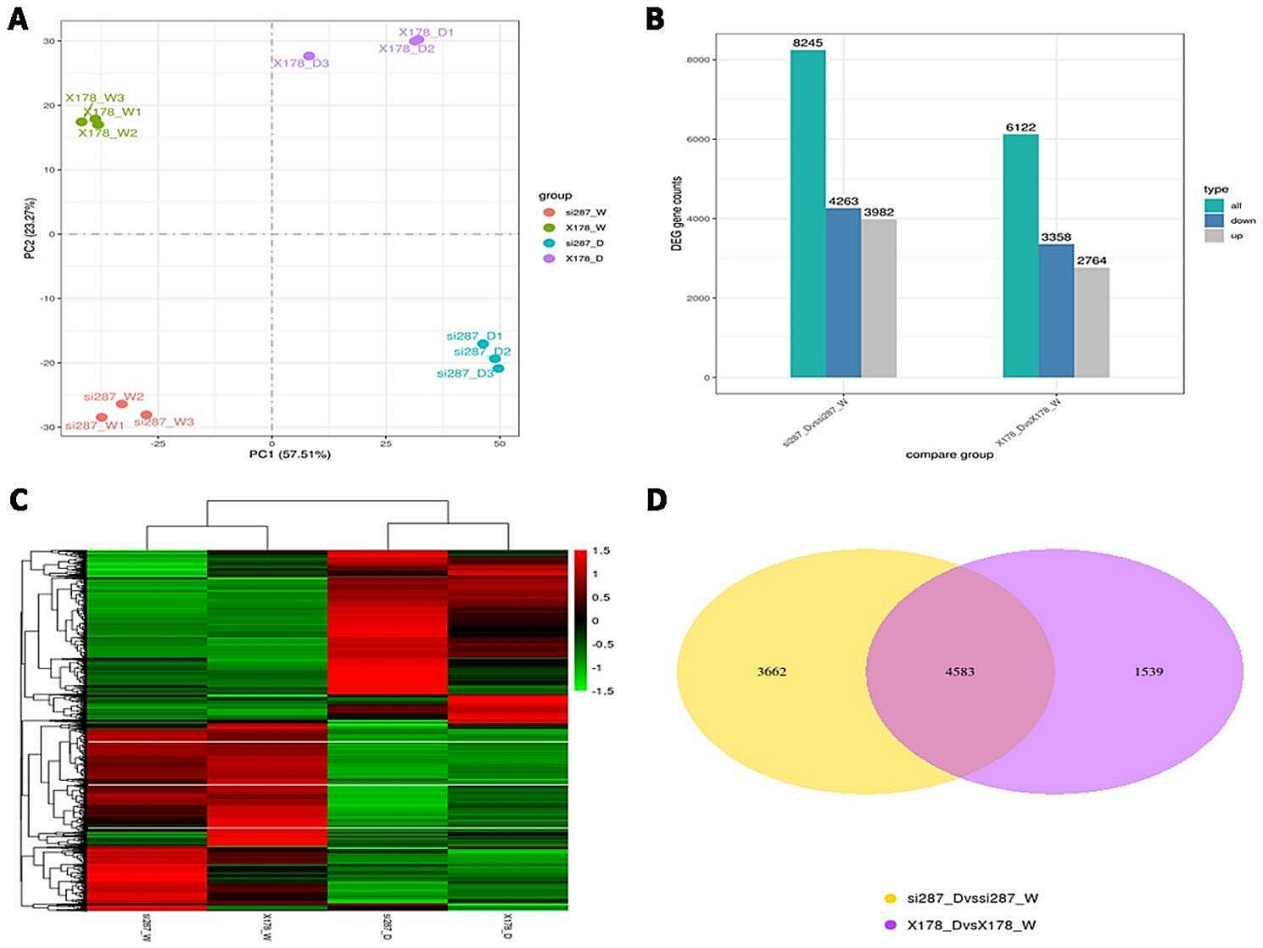
Overview of si287 and X178 transcriptome data after drought stress. (**A**), PCA of transcriptome samples. si287 and X178 were grown under well-water(W) and drought stress(D) conditions are indicated as different colour dots, respectively. (**B**), The number of totals, upregulated, and downregulated DEGs in the different comparison groups. (**C**), Clustering heatmap of DEGs. (**D**), Venn diagram of DEGs in the different comparison groups

To understand distinct responses of si287 and X178 to drought stress, we conducted a transcriptomic analysis of maize leaves from seedling plants exposed to D and W conditions. The high similarity among the three biological replicates within each treatment was confirmed by both the correlation coefficients (R^2^) and the principal component analysis of the transcriptomic data (Fig. 2A, Fig. S1). We identified a total of 8,245 (4,263 upregulated and 3,982 downregulated genes) DEGs in the si287 D vs. si287 comparison, and 6,122 DEGs (3358 upregulated and 2,764 downregulated genes) in the X178_D vs. X178 comparison (Fig. 2B), 4,583 DEGs overlapped between si287 and X178 (Fig. 2D). Interestingly, the number of upregulated DEGs in each comparison group was higher than the number of downregulated DEGs and the heatmap of DEGs between si287 and X178 was obviously different (Fig. 2C). These findings suggest that a considerable proportion of the transcriptomic changes in si287 and X178 subjected to drought stress resulted from upregulation of gene expression.

**Fig. 3:**
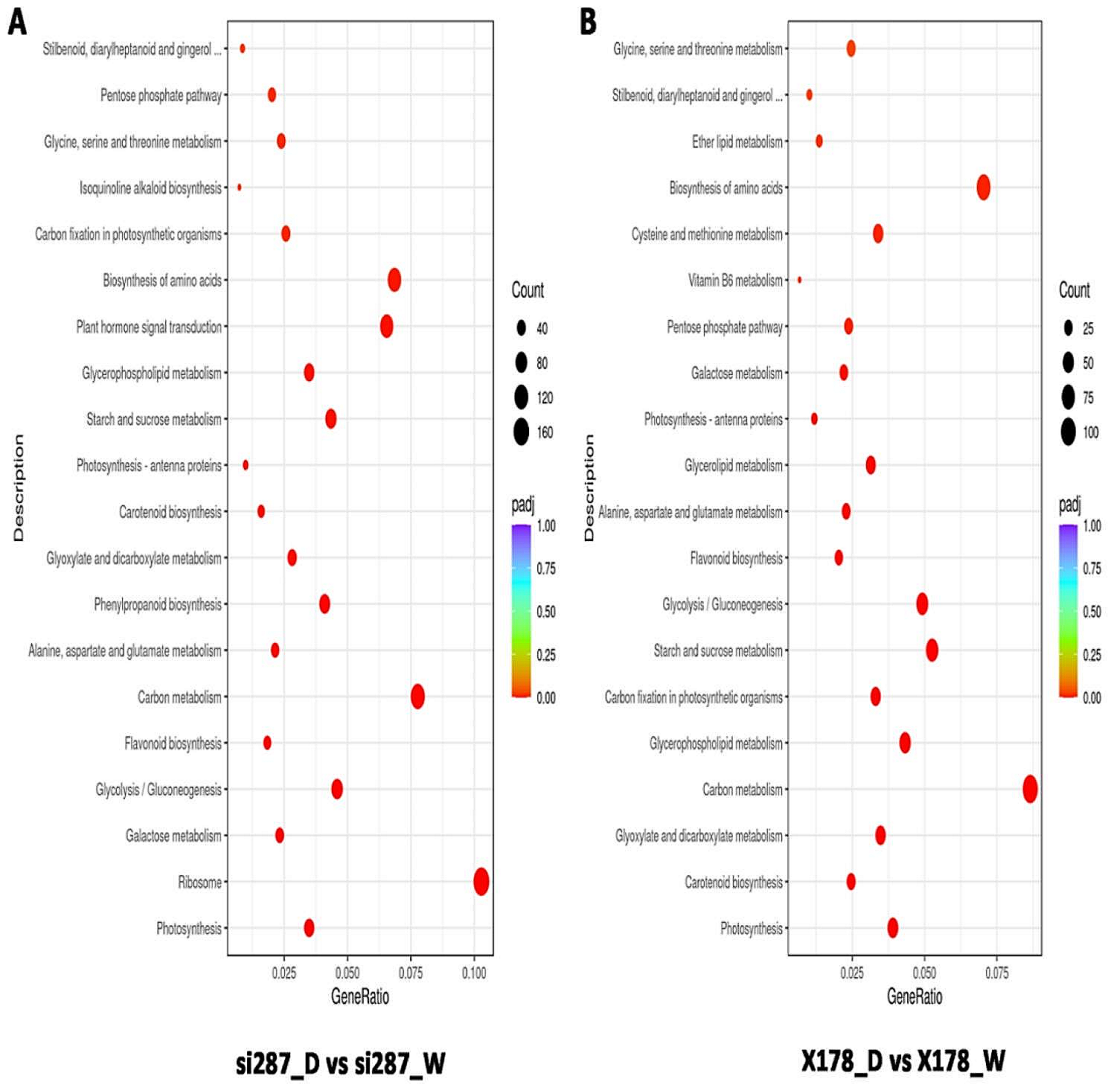
KEGG pathways enrichment analysis after drought stress treatment. (**A**) si287_D and si287_W; (**B**) X178_D and X178_W

The transcription characteristics of the drought-resistant inbred line si287 and the drought-sensitive inbred line X178 play a significant role in their response to drought stress. Therefore, we performed a functional enrichment analysis of DEGs in X178_D vs. X178 and si287_D vs. si287. Our analysis revealed that there was a significant enrichment of DEGs in photosynthesis (GO: 0015979) in si287 D vs. si287 in the biological processes (BP) category, while no significant enrichment was observed in X178_D vs. X178 (Table S2). In the molecular function (MF) category, the DEGs were significantly enriched in oxidoreductase activity (GO: 0016705), iron ion binding (GO: 0005506), tetrapyrrole (GO: 0046906) and hydrolase activity (GO: 0016798). In cellular component (CC) category, the DEGs were significantly enriched in thylakoid (GO:0009579) and photosystem (GO: 0009521) (Fig. S2; Table S2). These results imply that the identified GO terms play an essential role in the maize response to drought stress. Our analysis also revealed several KEGG pathways enriched in si287 compared to X178 under drought conditions. These pathways include glycolysis / gluconeogenesis (zma00010), flavonoid biosynthesis (zma00941), starch and sucrose metabolism (zma00500) and biosynthesis of amino acids (zma01230) (Fig. 3A, B; Table S2), indicating that these pathways contribute to the divergence of maize drought tolerance by regulating their activities under drought conditions.

Moreover, *ZmPP2C-A10, ZmGLK44* and *Bx12* are known as hub genes in maize drought tolerance [25, 26]. *ZmPP2C-A10* and *ZmGLK44* exhibited much higher expression level in si287 than in X178 between D and W conditions in our data. We only detected the expression of *Bx12* under D condition, and *s*imilarly, the expression level in si287 was significantly higher than that in X178 (Fig. S3, Table S3). These results further demonstrate the reliability of our analyses and provide clues for metabolic data analyses.

### Metabolic profiling of maize plants subjected to drought stress

**Fig. 4:**
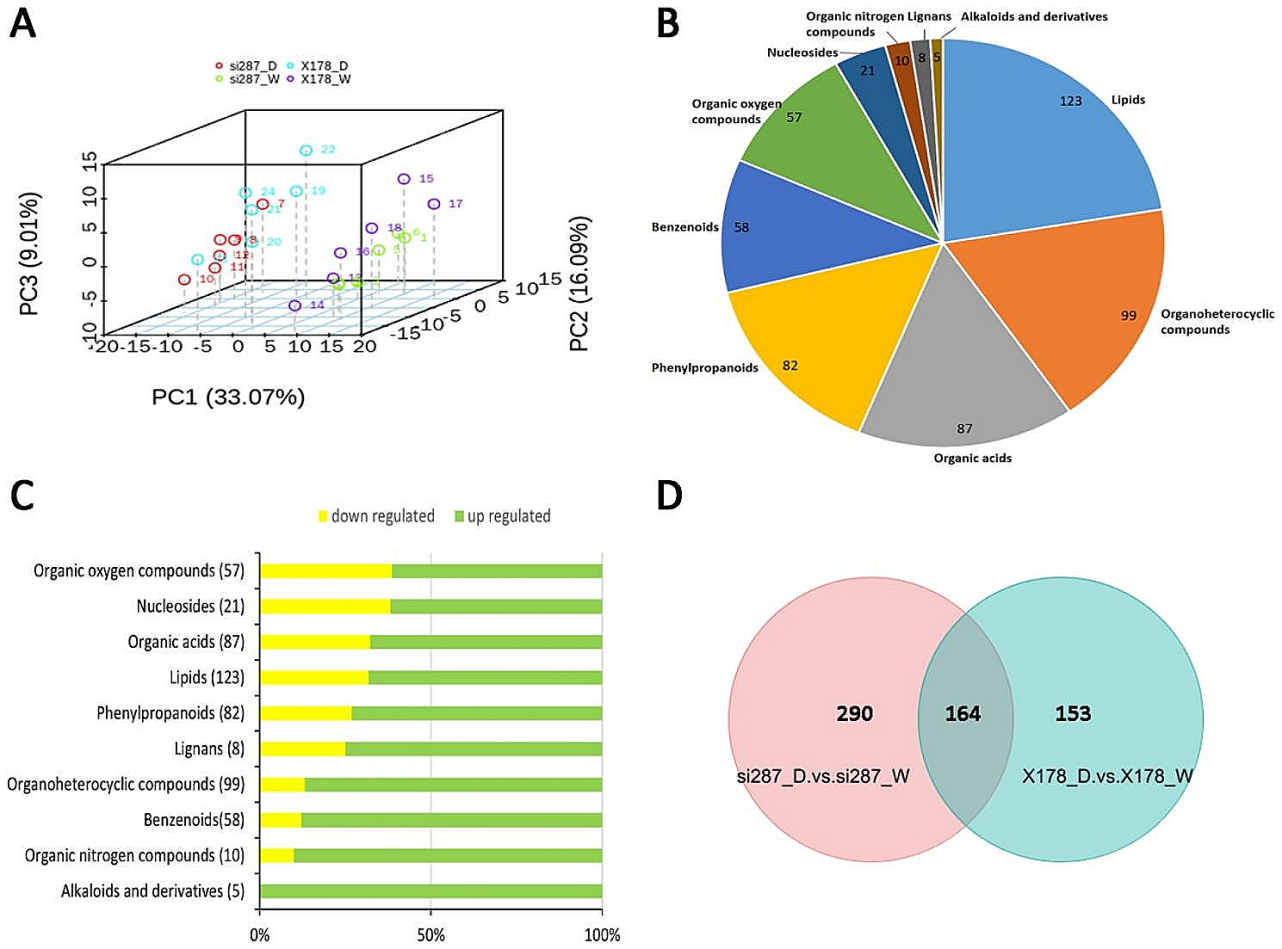
Overview of si287 and X178 metabolome data after drought stress. (**A**), PCA of si287 and X178 based on metabolites. si287 and X178 were grown under well-water(W) and drought stress(D) conditions are indicated as different colour dots, respectively. (**B**), Classes of the metabolites with HMDB annotations. (**C**), Proportions of the up- (green) and downregulated (yellow) metabolites with HMDB annotations. (**D**), Venn diagram of DEMs in the different comparison groups

To assess the impact of drought stress on metabolite production, we conducted PCA using all 1226 detected metabolites in si287 and X178 under D and W treatments. Our analysis implicated a clear separation between the W and D conditions, with principal component (PC) 1 explaining the largest proportion (33.07%) of the observed phenotypic variance (Fig. 4A). These findings indicated that leaf metabolites in si287 and X178 were extremely sensitive to drought stress. Our results are consistent with a previous study, which reported a significant influence of drought on metabolite production in maize seedlings [7]. To gain insights into the metabolic responses of maize to drought stress, we employed several assays to investigate changes in all detected 1,226 metabolites in si287 and X178 under W and D conditions. Using orthogonal partial least squares discriminant analysis (OPLS-DA), we found that 49.5% of the metabolite features contributed to drought response (VIP ≥ 607/1,226). Paired t-test showed that most metabolite features were significantly affected by drought stress, with 44.9% (550/1,226) showing notable changes in accumulation (|Fold Change| ≥ 2, false discovery rate [FDR] < 0.05). Notably, 74.3% (451/607) of the metabolites showed upregulation patterns in response to drought (Fig. 4C), revealing that upregulation of metabolites tends to play a dominant role in the regulation maize drought response, potentially conferring drought tolerance. Of the annotated metabolites, 350 were belonged to a broad range of classes, including lipids, organic acids, benzenoids and phenylpropanoids (Fig. 4B). 164 were overlapped between si287_D vs. si287_W and X178_D vs. X178_W (Fig. 4D).

**Fig. 5:**
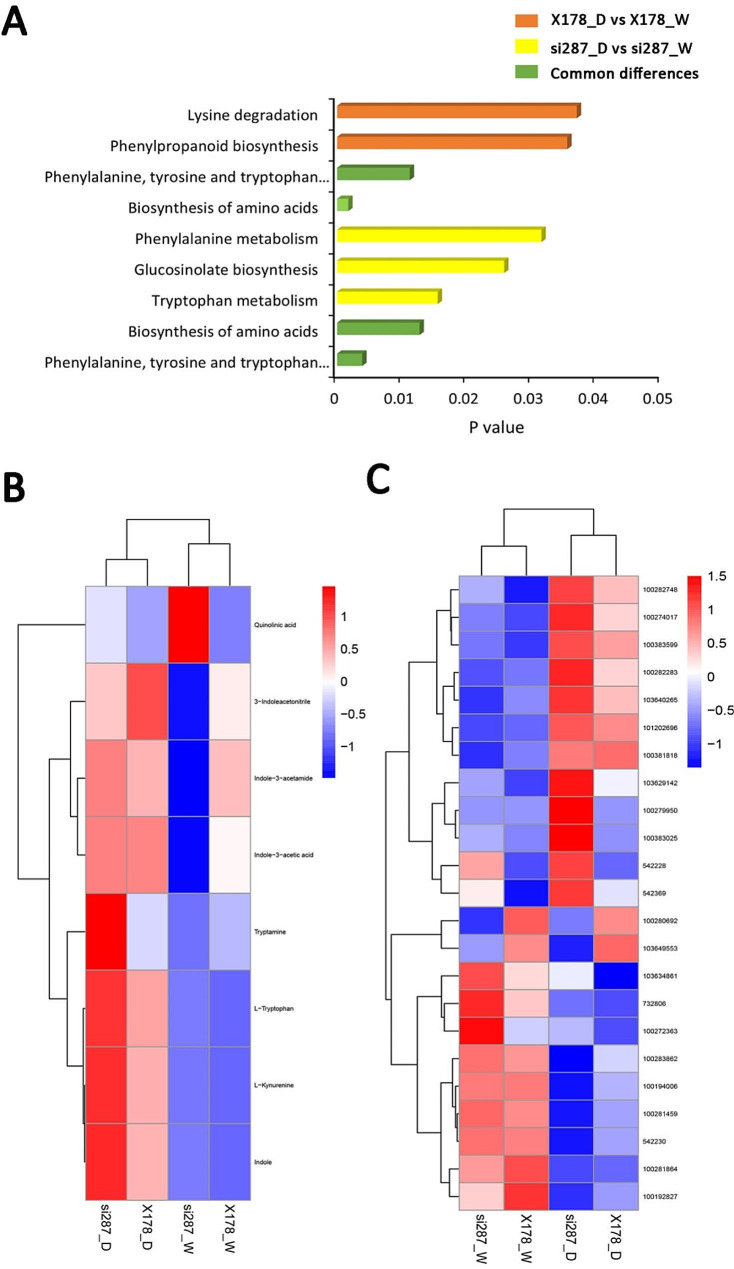
Activation of tryptophan (Trp) biosynthesis pathway genes and metabolites in response to drought stress. (**A**), Significantly enriched DEMs in KEGG pathways. Greens are the common different KEGGs in si287_D vs. si287_W and X178_D vs. X178_W. (**B**), Accumulations of Trp correlated metabolites in si287 and X178 plants under W and D conditions. (**C**), Trp correlated DEGs in si287 and X178 plants under W and D conditions

Furthermore, we carried out a KEGG enrichment analysis on the differentially expressed metabolites (DEMs). The results showed that DEMs were enriched in only 5 KEGGs of si287_D vs. si287_W, of which the significantly enriched pathways were phenylalanine, tyrosine and tryptophan biosynthesis (map00400), biosynthesis of amino acids (map01230), tryptophan metabolism (map00380), glucosinolate biosynthesis (map00966) and phenylalanine metabolism (map00360) (Fig. 5A; Table S4). In X178_D vs. X178_W, there were four enriched KEGGs, including two identical pathways with si287 comparison (phenylalanine, tyrosine and tryptophan biosynthesis (map00400) and biosynthesis of amino acids (map01230) (Fig. 5A), as well as phenylpropanoid biosynthesis (map00940), which is a central hub for the biosynthesis of defense-related metabolites [12], and lysine degradation (map00310) (Fig. 5A; Table S4). Together, these data indicated biosynthesis of amino acids, especially for phenylalanine, tyrosine, and tryptophan, is vital for maize to respond to drought stress.

### Regulation of amino acid biosynthesis pathway metabolites under drought stress

Regulation of amino acid biosynthesis pathway metabolites plays a crucial role in plant survival under drought stress. Proline is a key metabolite that accumulates rapidly in response to drought stress and is particularly important for osmoprotection [27]. Our metabolic profiling revealed that proline accumulated only in si287 D, indicating its importance in maize’s response to drought stress (Fig. 5A; Table S4). Another essential amino acid for plants to maintain access to water during drought stress is methionine, which is involved in root growth and development. Methionine also only accumulated in si287_D in our study [28, 29] (Fig. S4). Additionally, phenylalanine, tyrosine and tryptophan are only three aromatic amino acids among the 20 common amino acids. Increased contents of these 3 amino acids were associated with drought enhancing mechanisms, including stomatal regulation, plant hormone synthesis, and oxidative stress protection [30, 31]. We found that phenylalanine and tyrosine exhibited significance in both si287 and X178 under D condition compared to W condition (Fig. S5), showing their indispensability in response to drought stress. The phenylalanine ammonia-lyase genes were associated with oxidative stress protection as reported in a previous study [32]. Our KEGG analysis revealed that shikimic acid, which participates in salicylic acid (SA) synthesis and rapidly decreases after drought or heat stresses [33], was downregulated only in si287_D (Table S4). On the other hand, Trp KEGG was significantly upregulated only in si287_D vs. si287_W (Fig. 5A), suggesting a positive role of Trp in promoting maize survival under drought conditions. In general, these amino acids and related metabolites play critical roles in helping maize adapt and survive under drought stress. Further understanding of the gene regulation of these amino acids can aid in the development of strategies to enhance maize productivity under water-limited conditions.

### Exploring the role of genes and metabolites involved in Trp biosynthesis during seedling drought response

**Fig. 6:**
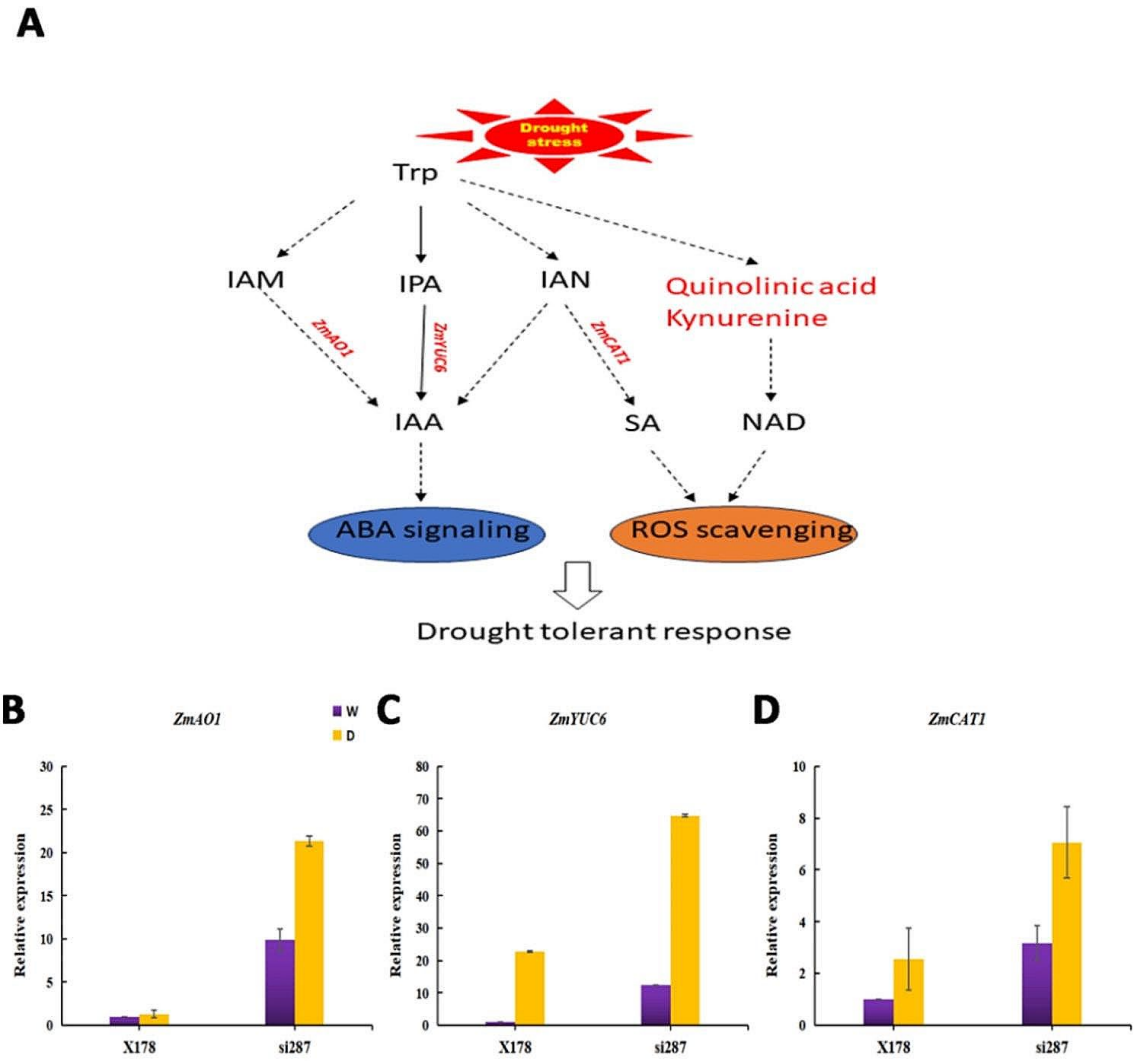
Key metabolites and expression of genes involved in Trp biosynthesis pathway. (**A**), A contextual summary of the Trp biosynthesis pathway involved in responding to drought stress in this study. Genes with red arrows represent the hub genes during drought stress; (**B**–**D**), Expression validation of hub genes in the Trp biosynthesis pathway. W represents well-watered control; D represents drought-stress treatment. ABA, abscisic acid; IAA, indole-3-acetic acid; IAM, indole-3-acetamide; IAN, indole-3-acetonitrile; IPA, indole-3-pyruvic acid; NAD, nicotinamide adenine dinucleotide; ROS Reactive oxygen species; SA salicylic acid

A previous study has revealed that Trp responds to water-deficient stress from early to late stages [34]. In this study, we analyzed all 23 genes enriched in tryptophan metabolism KEGG in si287_D vs. si287_W (*P* value = 0.00417, *P*_adj_=0.02155). Moreover, KEGG enrichment of tryptophan metabolism in X178_D vs. X178_W (*P* value=,0.05525 *P*_adj_=0.17771) did not reach a significant difference. Among the 23 genes, 19 (82.6%) genes are transcription factors (TFs) belonging to nine TF families. Thiolase_N TF members 101,202,696, 100,274,017, 100,282,283 and 103,640,265, as well as Pyr_redox_2 TF members 100,381,818 and 100,383,599 were upregulated after drought stress. *ZmAO1* (542,228) and *ZmCAT1* (542,369) were significantly upregulated only in si287_D (Figs. 5C and 6B and C). Flavin-containing monooxygenases (FMO)-like TF also known as *YUC* genes, which convert IPyA to IAA, were downregulated after drought stress [35–37]. They are Zm*YUC9* (100,281,459) and Zm*YUC5* (100,281,864). Nevertheless, Zm*YUC6* (103,629,142), another FMO-like TF member, was strongly upregulated after drought stress, especially in si287_D (Figs. 5C and 6D). Zm*YUC6* was reported to increase endogenous IAA levels with drought tolerance compared to wild-type plants in *Arabidopsis thaliana* [38]. According to the clustering heatmap, the most abundant DEMs of tryptophan metabolism were quinolinic acid, tryptamine, L-tryptophan, kynurenine and indole. Quinolinic acid decreased rapidly after drought stress. Tryptamine, L-tryptophan, kynurenine, and indole were more abundant in si287_D than in X178_D (Fig. 5B), with indole identified as a key intermediate in the synthesis of tryptophan [39].

## Discussion

In recent years, the rapid developments of high-throughput RNA sequencing and metabolite profiling have enabled the identification of numerous metabolites and their regulated genes in various crops grown under normal conditions [40–42]. In this paper, we uncovered novel genes and metabolites in tryptophan biosynthesis pathway associated with the response to drought stress during maize seedlings stage. These findings have the potential to enhance our comprehension of the molecular mechanisms underlying the drought stress response in maize and other crops.

### KEGG of transcriptomics and metabolomics to elucidate mechanisms of maize seedling responses to drought stress

KEGG is a powerful tool that enable a comprehensive understanding of the enrichment of DEGs and DEMs. Our KEGG analysis provided strong clues to illustrate maize seedling responses to drought stress. However, there are some queries that need to be addressed. Our KEGG result did not reveal enrichment in pathways related to ROS, ABA biosynthesis and signaling, in contrast to the transcriptomic and metabolomic analyses of *Selaginella tamariscina* [43, 44]. This plant species is considered an ideal model for exploring the genetic basis of plant drought tolerance [45]. Nevertheless, it is noteworthy that tryptophan serves as a precursor in several pathways associated with IAA-ABA signaling and ROS scavenging. Our results appear to be positioned further upstream in these pathways. We identified flavonoid biosynthesis KEGG enriched in transcriptome data, while we did not detect KEGG enrichment in metabolome data. Only several flavonoid biosynthesis related metabolites showed significant differences in content after drought stress. They are naringin, (+)-catechin and neohesperidin (Figs. S4 and S5). Reasons for the absence of enriched flavonoid biosynthesis KEGG in the metabolome data could be the limited accuracy of metabolite detection. Some small molecules may not have been detected. Further studies are needed to bridge the gap between differences in gene expression and metabolite contents.

### Role of tryptophan metabolism in maize’s response to drought stress during seedling stage

Amino acid metabolism is critical for various cellular processes such as defense and signaling [46]. The metabolism of tryptophan is significantly affected by drought stress. The biosynthesis and degradation of tryptophan is related to glycolysis/gluconeogenesis or the TCA cycle. *ZmCS1* (citrate synthase 1, 100,280,203) significantly displayed downregulation after drought stress (Fig. S3), which is a key gene involved in the TCA cycle associated with phenylpropanoid hydroxycitric acids, flavonoids and benzoxazinoids [10]. Moreover, our study also examined the expression of several genes related to glycolysis/gluconeogenesis, including *ZmFBP* (100,273,700), *ZmPGM2*(542,358), *ZmHXK1* (100,192,075) and *ZmHXK2* (100,283,735) [25]. We found that *ZmFBP* and *ZmPGM2* were significantly downregulated after drought stress, while *ZmHXK1* and *ZmHXK2* displayed strong upregulation after drought stress (Fig. S3).

Tryptophan, an essential amino acid, is crucial for the growth and development of plants. It plays an essential role in various metabolic processes within the plant, including the production of hormones (IAA, ABA, and SA), pigments (**c**hlorophyll and flavonoids) and secondary metabolites (alkaloids and terpenoids). These components are critical for plants to protect themselves from abiotic stresses and diseases. However, previous studies have focused more on IAA, ABA, and flavonoids in maize responses to drought stress, neglecting the importance of tryptophan in synthesizing these metabolites, which plays a significant role in drought stress responses (Fig. 6A, Fig. S6). Our transcriptomics data also showed that the expression level of *ZmGLK44*, a TF regulating Trp biosynthetic, increased 5.9 times in X178 and 23.8 times in si287 after drought stress [25]. We further confirmed the involvement of *SnRK2.4*, *ZmPP2C-A4, ZmPP2C-A7*, *ZmRD17*, *ZmRAB18*, and *ZmLEA* in the ABA signaling pathway activating by ZmGLK44 by Real-time PCR (Fig. S7).

Furthermore, we conducted joint transcriptomics and metabolomics analysis to study the metabolites and related genes of tryptophan biosynthesis. Three novel hub genes, namely *ZmAO1* (542,228), *ZmCAT1* (542,369) and Zm*YUC6* (103,629,142), exhibited significant upregulation in response to drought stress in si287_D (Fig. 5C). The enhanced expression of *ZmAO1* and Zm*YUC6* suggests they enhanced drought tolerance of maize through auxin signal pathway, whereas *ZmCAT1* appears to contribute to drought tolerance through SA pathway. Shikimic acid, the SA precursor, demonstrated a decrease accumulation in si287_D (Fig. 6A; Fig. S6). Quinolinic acid and kynurenine are critical for NAD biosynthesis and actively contribute to the drought response by scavenging ROS. Notably, a coding quinolinate phosphoribosyl transferase gene (100,382,929) displayed negative accumulation after drought stress (Figs. 2C, 5B and 6A). These approaches have provided insights into the primary reactions to drought stress, revealing the novel genes and metabolites crucial in maize. Furthermore, our study suggests the possibility of using Trp to enhance drought tolerance for maize plants during the seedling stage (Fig. 6; Fig. S3; Fig. S8). This approach could lead to the development of new strategies for enhancing crop yield and mitigating the effects of drought stress on agriculture.

## Conclusions

Our analyses of transcriptomics and metabolomics data of maize drought-sensitive line X178 and drought-tolerant line si287 have provided novel insights into the primary reactions to drought stress in maize seedling plants. The factors contribute to the divergence of maize drought tolerance: (1) glycolysis/gluconeogenesis, flavonoid biosynthesis, starch and sucrose metabolism and biosynthesis of amino acids pathways. (2) proline, methionine, and tryptophan amino acids. (3) quinolinic acid and kynurenine (4) *ZmAO1*(542,228), *ZmCAT1*(542,369) and *ZmYUC6* (103,629,142) are the hub genes.

Our findings highlight the crucial role of tryptophan in the maize response to drought stress through its involvement in IAA-ABA signaling, as well as SA and NAD consequent ROS scavenging pathways. Additionally, genes and metabolites closely related to tryptophan biosynthesis are potential targets for genome editing and other biotechnological strategies to improve maize tolerance and yield under drought stress.

### Electronic supplementary material

Below is the link to the electronic supplementary material.

**Supplementary Material 1:** Supplementary Figs. S1–S8 and Supplementary Table 1

**Supplementary Material 2:** Significant enrichment (*P*_adj_<0.05) of GO and KEGG pathways in transcriptomic data

**Supplementary Material 3:** The expression data of key genes after drought treatments

**Supplementary Material 4:** Significant enrichment (*P*_adj_<0.05) of KEGG pathways in metabolomic data

**Supplementary Material 5:** Primers for Real-time PCR

## Data Availability

The datasets presented in this study can be found in online repositories. The names of the repository/repositories and accession number(s) can be found below: NCBI accession number: PRJNA952945.

## Abbreviations

ABA: Abscisic acid
DEGs: Differentially expressed genes
DEMs: Differentially expressed metabolites
FPKM: Fragments per kilobase of exons per million mapped fragments
GO: Gene ontology
KEGG: Kyoto encyclopedia of genes and genomes
LC-MS: Liquid chromatography-mass spectrometry
NAD: Nicotinamide adenine dinucleotide
PCA: Principal component analysis
ROS: Reactive oxygen species
RWC: Relative water content
SA: salicylic acid
TF: Transcription factor
VIP: Variable importance for the projection
