# Supplementary material for: Utilizing transcriptomics and metabolomics to unravel key genes and metabolites of maize seedlings in response to drought stress
Source: BMC Plant Biol. 2024 Jan 8;24:34. doi: 10.1186/s12870-023-04712-y (PMC10773024; doi:10.1186/s12870-023-04712-y)
Supplement: Supplementary file 1 — Supplementary Material 1: Supplementary Figs. S1–S8 and Supplementary Table 1 [file 12870_2023_4712_MOESM1_ESM.docx]

**Supplementary Material**

**
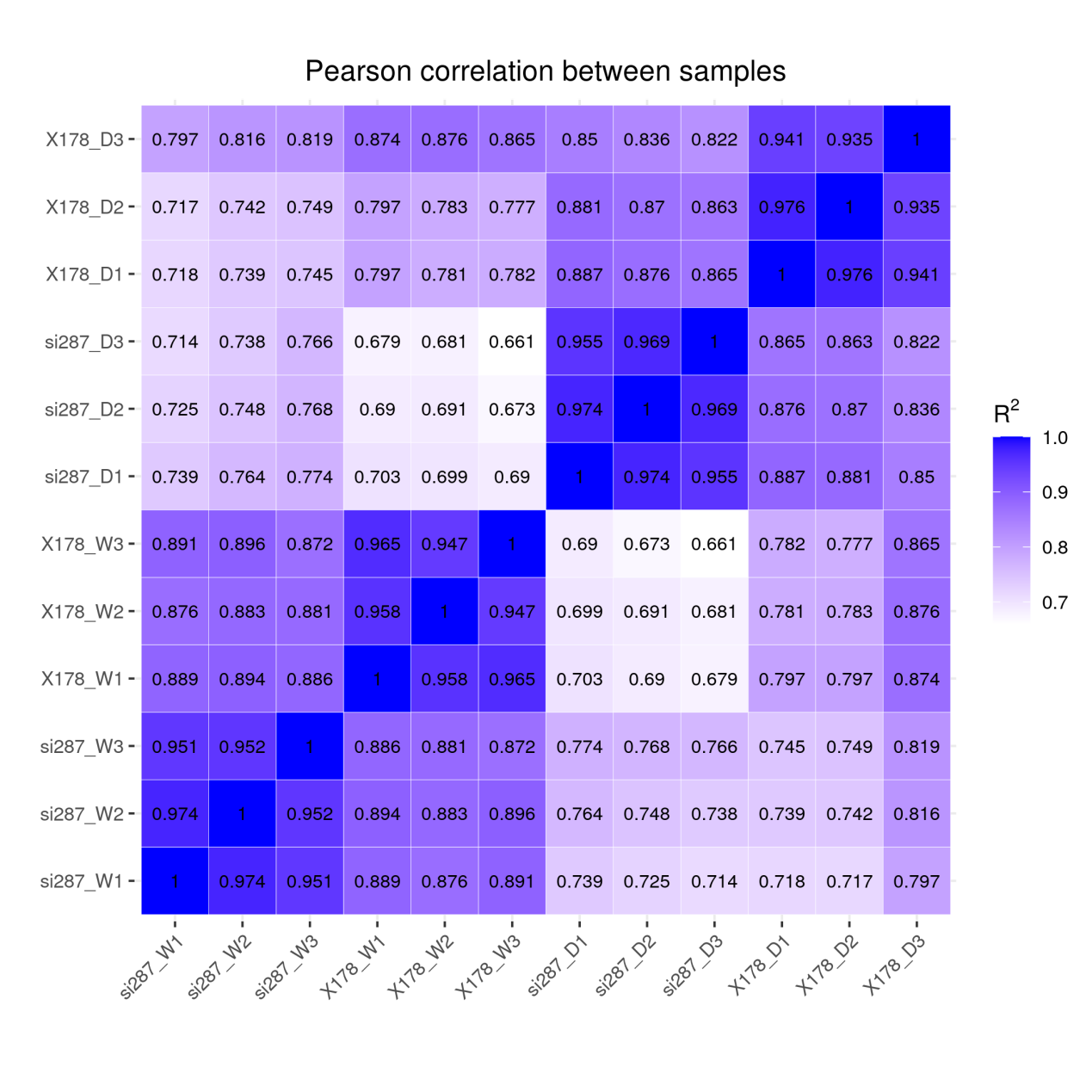
**

**Fig. S1** Pearson correlation between transcriptome samples.


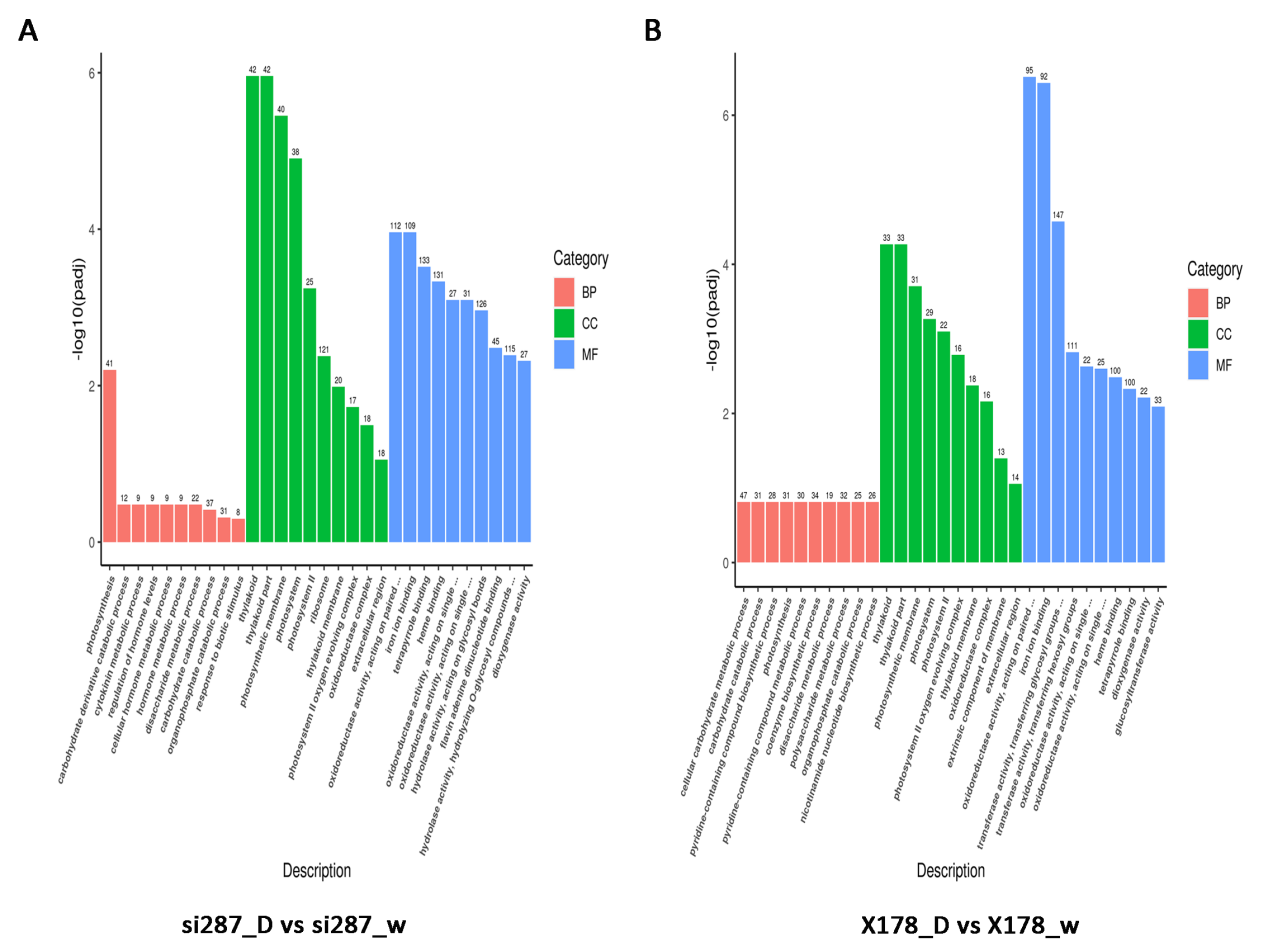


**Fig. S2** GO enrichment analysis after drought stress treatment. (A) si287 D and W; (B) X178 D and X178 W.


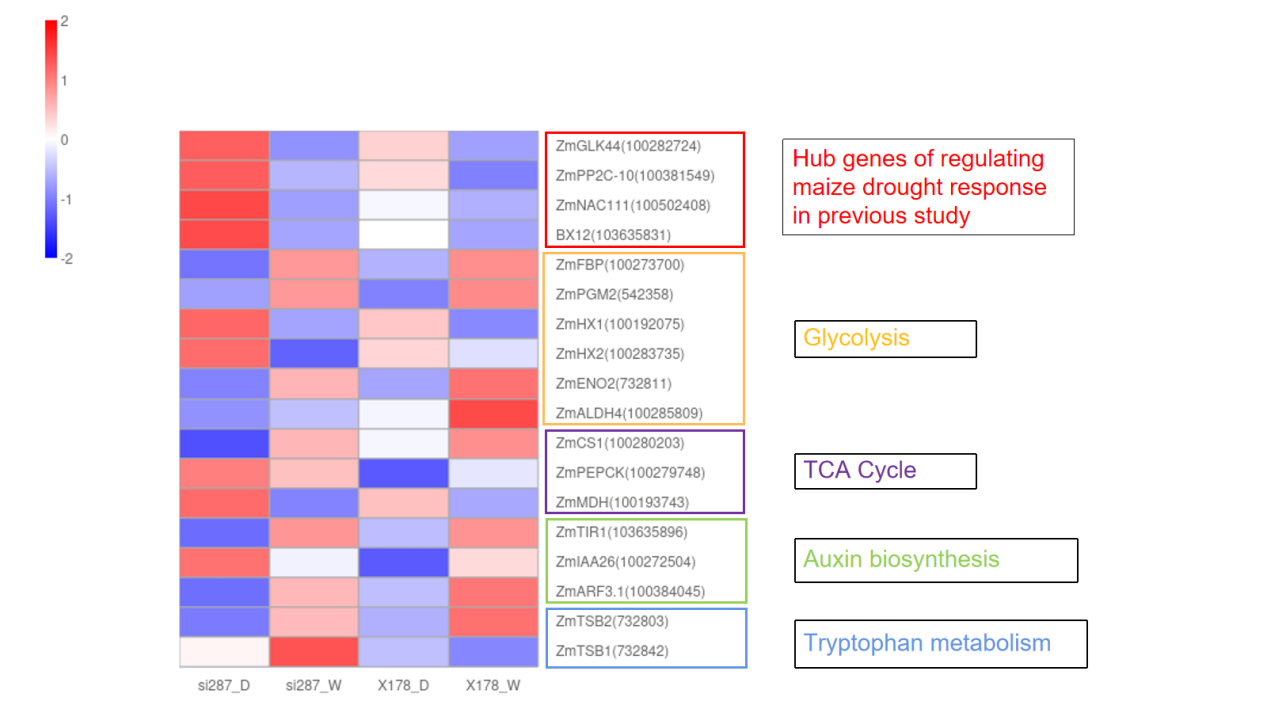


**Fig. S3** The expression of key genes involved in drought response under W and D treatments. Red indicated genes with high expression level.

**
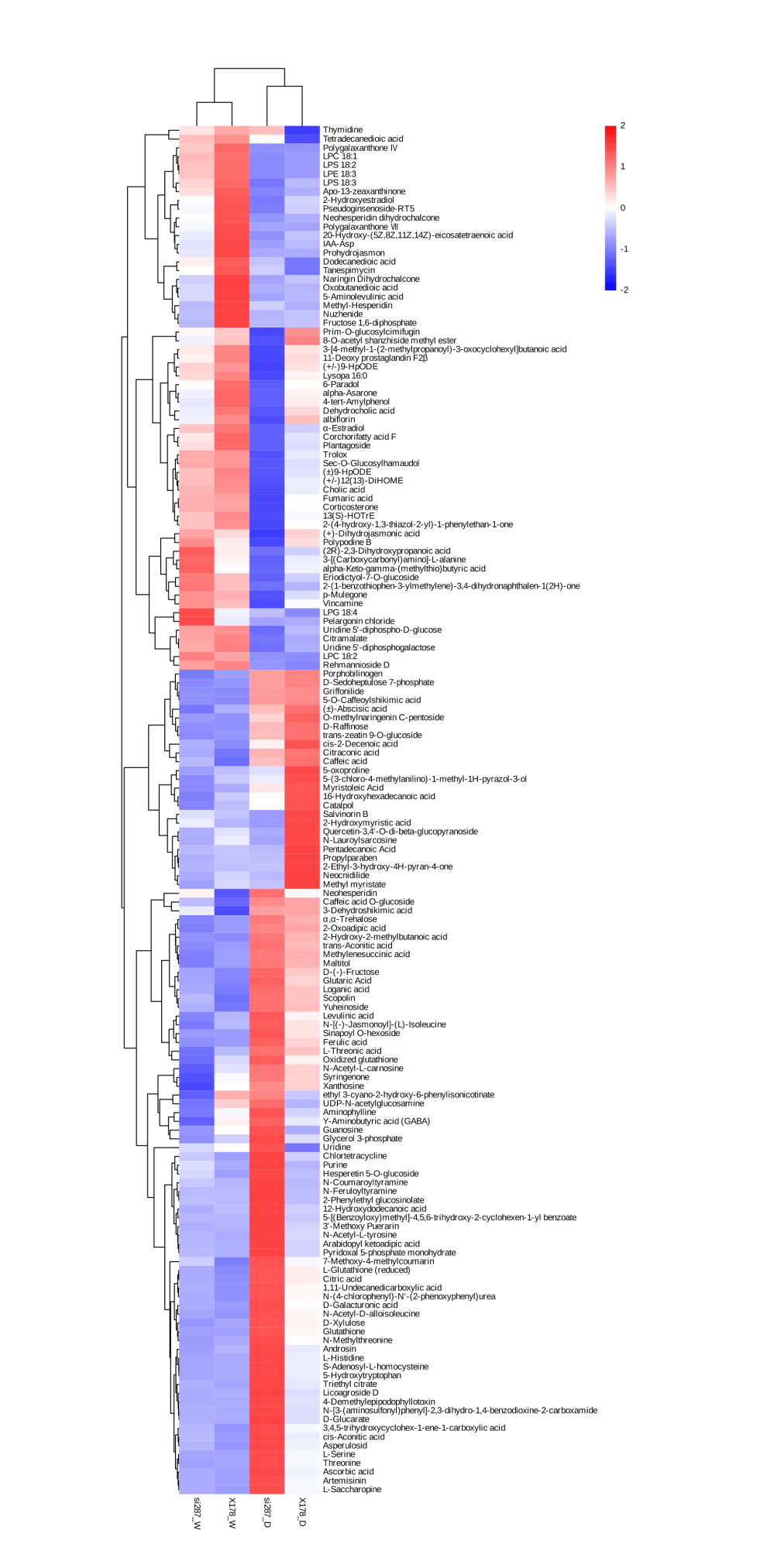
**

**Fig. S4** Heatmap showing the differential abundance of negatively charged metabolites in response to drought stress.

**
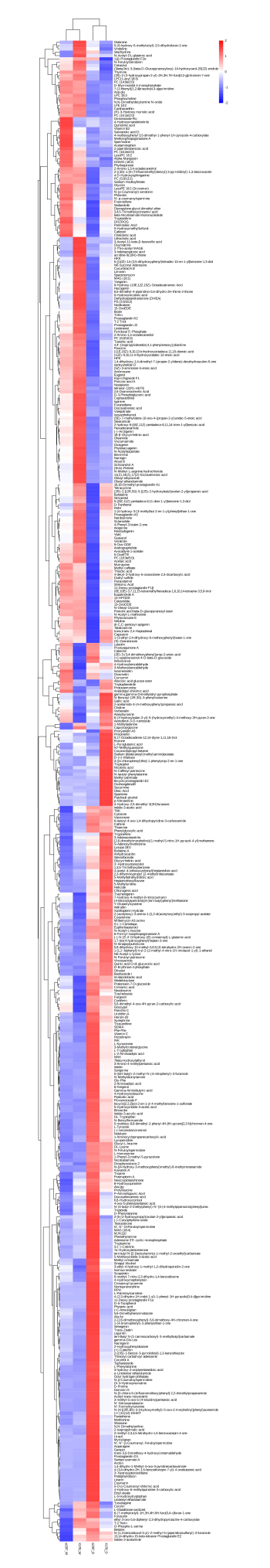
**

**Fig. S5** Heatmap showing the differential abundance of positively charged metabolites in response to drought stress.


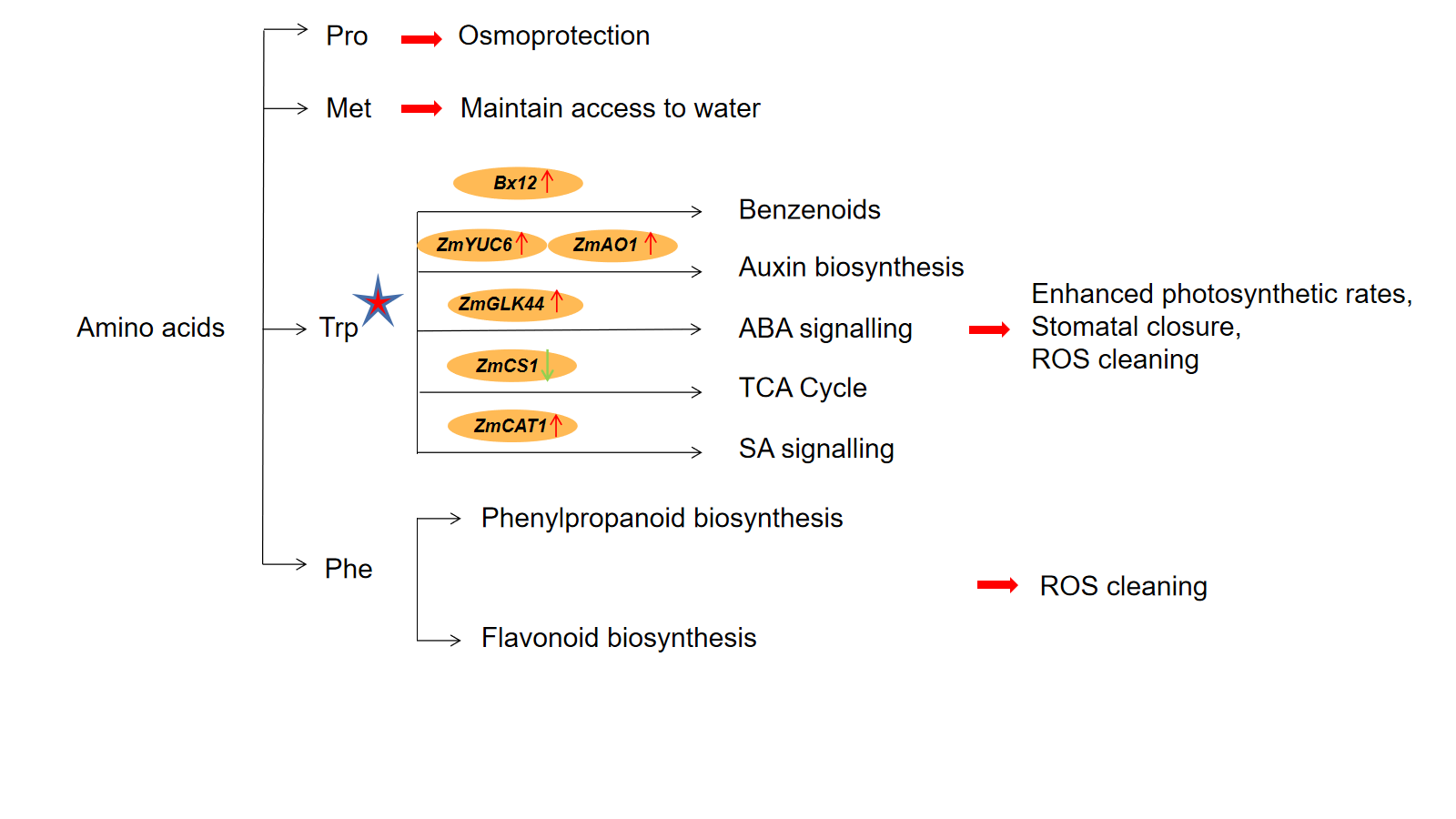


**Fig. S6** A contextual summary of the amino acids involved in responding to drought stress in this study. Genes with red arrows represent upregulated after drought stress; the gene with green arrow represents downregulated after drought stress.


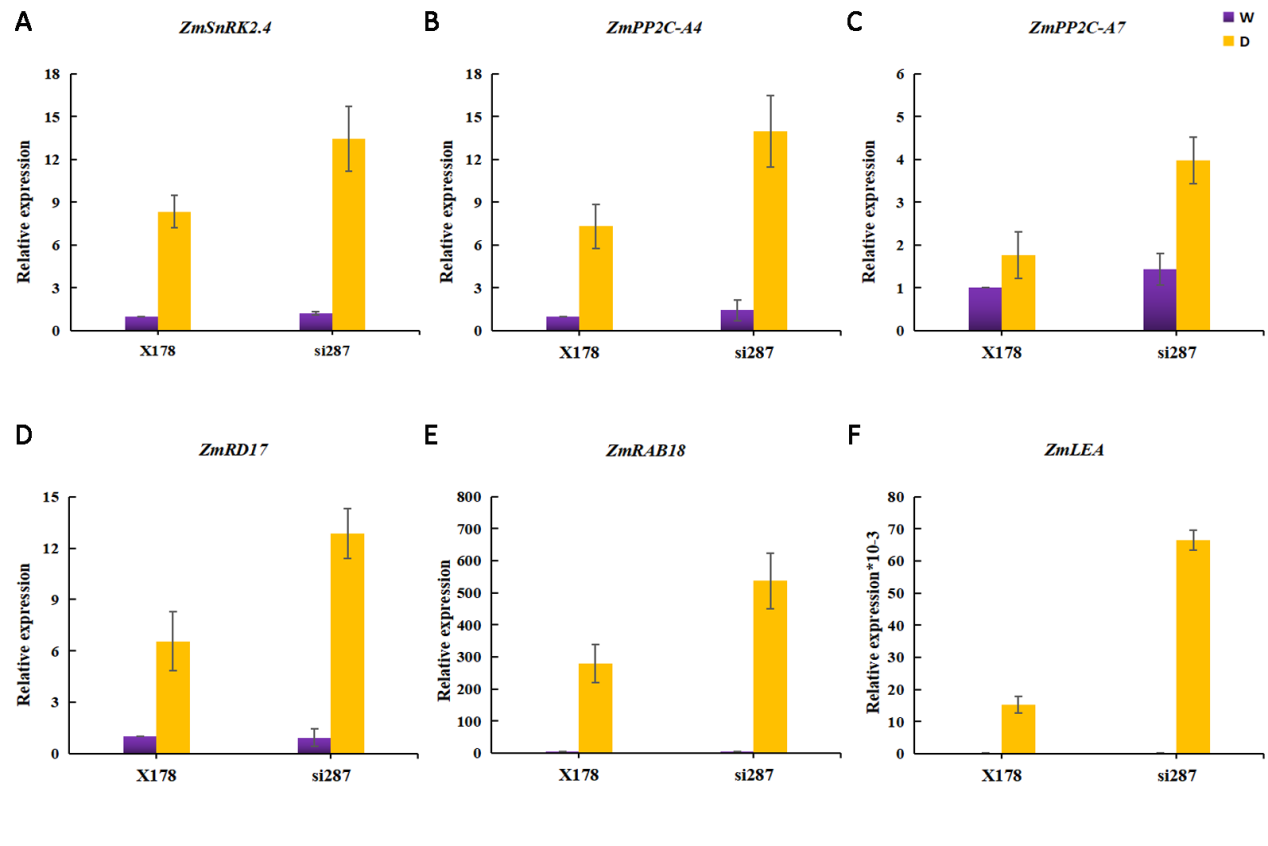
**Fig. S7** Expression validation of ABA responsive genes. Real-time PCR was performed to validate the expression of ABA responsive genes in the si287 and X178 under W and D conditions, including *ZmSnRK2.4* (100384302), *ZmPP2C-A4* (100279578), *ZmPP2C-A7* (100381666), *ZmRAB18* (542373), *ZmRD17* (100281087) and *ZmLEA* (100283639).


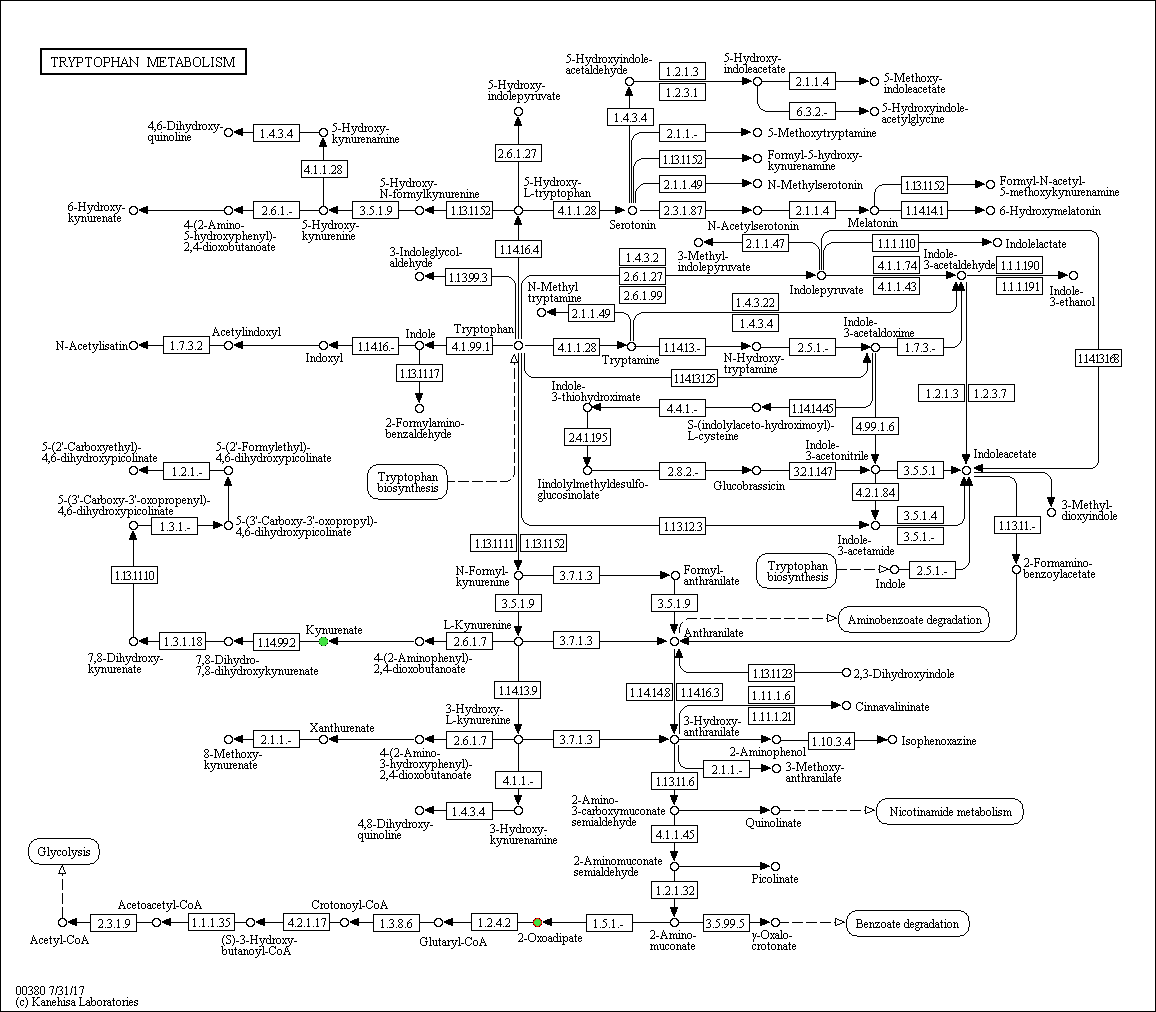


**Fig. S8** Tryptophan metabolism pathway

**Supplementary Tables**

**Table S1** Dry weight traits performance of si287 and X178 under drought stress

| Treatments | No. of plants | Shoot dry weight(g) | Root dry weight（g） | Total dry weight（g） | Relative biomass |
| --- | --- | --- | --- | --- | --- |
| X178_W | 12 | 0.62 | 0.17 | 0.79 | 0.8167±0.0352 |
| X178_D | 12 | 0.51 | 0.14 | 0.65 |  |
| si287_W | 12 | 0.46 | 0.13 | 0.59 | 0.8977±0.0194 |
| si287_D | 12 | 0.42 | 0.11 | 0.53 |  |

**Table S2** Significant enrichment (*P*_adj_<0.05) of GO and KEGG pathways in transcriptomic data

**Table S3** The expression data of key genes after drought treatments

**Table S4** Significant enrichment (*P*_adj_<0.05) of KEGG pathways in metabolomic data

**Table S5** Primers for Real-time PCR
